# Nomogram to predict the incidence of delirium in elderly patients with non-severe SARS-CoV-2 infection

**DOI:** 10.3389/fpsyt.2023.1288948

**Published:** 2024-01-11

**Authors:** Guanghui An, Zhihua Mi, Dongmei Hong, Dandan Ou, Xiaoxiao Cao, Qidong Liu, Lize Xiong, Cheng Li

**Affiliations:** Department of Anesthesiology and Perioperative Medicine, Shanghai Key Laboratory of Anesthesiology and Brain Functional Modulation, Clinical Research Center for Anesthesiology and Perioperative Medicine, Translational Research Institute of Brain and Brain-Like Intelligence, Shanghai Fourth People's Hospital, School of Medicine, Tongji University, Shanghai, China

**Keywords:** nomogram, delirium, SARS-CoV-2, Omicron, elderly patients, non-severe infection

## Abstract

**Objective:**

To construct and validate nomogram models that predict the incidence of delirium in elderly patients with non-severe SARS-CoV-2 infection.

**Methods:**

Elderly patients (≥65y) tested positive for SARS-CoV-2 infection at the hospital were included. We used the 3-min diagnostic Confusion Assessment Method for delirium diagnosis. Least absolute shrinkage and selection operator (LASSO) logistical regression analysis was performed to explore potential independent influencing factors of delirium. A predict model visualized by nomogram was constructed based on the confirmed variables. The predictive accuracy and clinical value of the model were evaluated using receiver operating characteristic (ROC) curves.

**Results:**

The data of 311 elderly patients were analyzed, of whom 73 (23.47%) patients were diagnosed with delirium. Three independent influencing factors of delirium were confirmed: age (OR1.16,1.11–1.22), Glomerular filtration rate (OR 0.98,0.97–0.99), platelet-large cell ratio (1.06,1.02–1.10). These parameters were used to create a nomogram to predict the development of delirium, which showed good predictive accuracy confirmed by the ROC curves (AUC 0.82,0.76–0.88).

**Conclusion:**

We construct a credible nomogram to predict the development of delirium in elderly patients with Non-severe SARS-CoV-2 infection. Our finding may be useful to physicians in early prevention and treatment of delirium.

## Introduction

COVID-19, caused by severe acute respiratory syndrome coronavirus 2 (SARS-CoV-2), has wreaked havoc worldwide for over 2 years. As of September 11, 2022, there have been 605 million confirmed cases and 6.4 million deaths (1). It is a major global public health challenge (2), especially after the B.1.1.529 (Omicron) variant appeared. The Omicron variant has quickly raised serious concerns globally owing to its enhanced transmissibility, rapid spread (3), and immune evasion (4). In late February 2022, a wave of SARS-CoV-2 infection appeared in Shanghai, China. According to the Shanghai Municipal Health Commission, as of May 4, 2022, over 600,000 cases had been identified; all new viral genomes in Shanghai were of the Omicron variant (5). Although the Omicron variant was highly infectious, its pathogenicity had decreased (6). Severe illness caused by Omicron infection was much lower than caused by the Delta variant (7).

Delirium, characterized by acute changes in attention and cognitive function, is a common complication among elderly patients in hospitals (8). It has been reported throughout the course of COVID-19, especially in elderly patients with severe SARS-CoV-2 infection (9, 10). The incidence of delirium among patients with COVID-19 is 11–65%, due to differences in assessment methods and demographic groups studied (9–12). Delirium can have a dramatic impact on elderly patients, even on mortality; therefore, its prevention and early diagnosis are important (8, 13).

The pathogenicity of Omicron is relatively lower that previous variants (7). The proportion of patients with severe disease has reduced, but the number of infected people is quite large, and a considerable majority of them are elderly. Studies also showed that mild respiratory SARS-CoV-2 infection can cause multi-lineage cellular dysregulation and myelin loss in the brain (14) and may lead to long-term cognitive decline (15). However, few studies have focused on the incidence of delirium, and potential related clinical characteristics, in the millions of patients with non-severe Omicron infection. However, delirium is also easy to be misdiagnosed, while most of the delirium hypoactive and quite (16), and there is no targeted treatment, so early prevention and diagnosis of delirium is particularly important, especially now that the number of elderly people infected with Omicron is increasing. We aimed to construct and validate nomogram models that predict the incidence of delirium in elderly patients with non-severe SARS-CoV-2 infection.

## Methods

### Study design, setting, and population

This is a prospective observational clinical study. The protocol was reviewed and approved by the Medical Ethics Committee of Shanghai Fourth Hospital (certificate 2022063–001). The study was registered at the Chinese Clinical Trial Registry Center (No. ChiCTR2200058903). All procedures were performed in accordance with the principles of the Declaration of Helsinki. All patients provided written informed consent before recruitment.

The study was performed in Shanghai Fourth People’s Hospital, a designated hospital during the Omicron pandemic in Shanghai since April 10, 2022. The patients admitted to the hospital between April 19 and June 6, 2022, were involved recruited if nasopharyngeal samples were positive for SARS-CoV-2 by reverse transcriptase–polymerase chain reaction (RT-PCR).

The inclusion criteria were as follows: age of ≥65 years, admission to our hospital for the first time with mild SARS-CoV-2 infection, corrected visual acuity and hearing within normal limits, agreed and cooperated to complete the assessment, and provided written informed consent. The exclusion criteria were admission to a cabin mobile hospital or any other designated hospital because of COVID-19, severe cases admitted to the ICU, mental illnesses, previous neurological diseases (such as dementia, craniocerebral tumor, cerebral hemorrhage, and cerebral infarction) according to medical history, severe defects of corrected vision or hearing, inability to complete the assessment, and patient refusal.

### Data collection

We performed the 3-min diagnostic Confusion Assessment Method (3D-CAM) to diagnose delirium. The first assessment should be completed within 24 h of ward admission by two of our doctors. Assessment was performed twice a day until the patient was discharged or diagnosed with delirium. Delirium was also recorded if the nurse or doctor-in-charge reported the event. We collected patient data including age, sex, body mass index (BMI), symptoms, number of long-term medications, number of vaccine doses against COVID-19, the first laboratory tests and chest computed tomogram, days to the outcome (i.e., days in-hospital or days from admission to delirium), and medications to treat COVID-19. We observed the incidence of delirium and analyzed factors affecting the development of delirium.

### Analysis

All the baseline information age, sex, body mass index (BMI), symptoms, number of long-term medications, number of vaccine doses against COVID-19 and the first laboratory tests and chest computed tomogram, days to the outcome of the including patients are described. Patients are divided into two groups, delirium group including patients who developed delirium and control group including patients who did not develop delirium. The measurement variables are shown using mean ± standard deviation (SD) while the distribution of the variable is normal distribution. The comparison between measurement variables of normal distribution were conducted using the *t*-test. If not, the median and interquartile range, (IQR) are used, and the comparison of non-normal distribution variables are analyzed using non-parametric test. Frequency and percentage are used to describe the count variables. The comparison between them is analyzed by Chi-square test, adjusted Chi-square test or Fisher exact test. When the *p* value <0.05, the comparison is significant. Using Cross-Validation by Least absolute shrinkage and selection operator (LASSO, using glmnet 4.1–2 package on R 4.0.5.) logistical regression to screen variables which is significantly different between groups. Univariate and multivariate logistic regression analyses were used to evaluate the effects of the selected variables on the development of delirium. Then a predicting model visualized by nomogram was constructed based on confirmed variables. The predictive accuracy and clinical value of the nomograms were evaluated using receiver operating characteristic (ROC) curve and the Decision Curve Analysis (DCA, using rmda 1.6 package on R 4.0.5.), respectively.

## Results

Between April 19 and June 6, 2022, 1,559 patients were admitted to our ward on the 7th–19th floors of our hospital because of Omicron infection. Of these, 316 patients met the inclusion criteria. However, five were excluded; two developed new cerebral infarction, and three progressed to severe COVID-19. Finally, 311 patients (131 males, 180 females; age, 75 ± 7 years) were included in the analysis; 73 (29 males, 44 females) were diagnosed with delirium, yielding an incidence of 23.47%. Table 1 summarizes the baseline characteristics of the two groups.

**Table 1:** Summarizes the baseline characteristics of the patients in two groups.

	No-delirium	Delirium	*p* value
	*N* **=** 238	*N* = 73	
Age (y)	73.00 [69.00, 79.00]	84.00 [76.00, 86.00]	<0.001
Gender (%)			0.735
Male	102 (42.9)	29(39.7)	
Female	136 (57.1)	44 (60.3)	
BMI (kg/m^2^)	22.75 [20.70, 24.30]	22.75 [21.90, 24.60]	0.141
Days to outcome (days)^1^	8.00 [5.00, 11.00]	5.00 [4.00, 8.00]	<0.001
Medical history			
Hypertension	120 (50.4)	42 (57.5)	0.352
Diabetes	39 (16.4)	13 (17.8)	0.916
Coronary	32 (13.4)	11 (15.1)	0.875
Pulmonary disease	20 (8.4)	4 (5.5)	0.570
CT reports (%)			0.478
Normal	74 (31.1)	21 (28.8)	
Pneumonia	85 (35.7)	21 (28.8)	
Pulmonary interstitial hyperplasia	57 (23.9)	21 (28.8)	
Pneumonia+Pulmonary interstitial hyperplasia	22 (9.2)	10 (13.7)	
Treat (%)			0.026
Chinese traditional medicine	37 (15.5)	14 (19.2)	
Anti-coronavirus drugs	51 (21.4)	6 (8.2)	
Chinese traditional medicine+ Anti-coronavirus drugs	143 (60.1)	53 (72.6)	
None	7 (2.9)	0 (0.0)	
First symptom			
Cough	122 (51.3)	36 (49.3)	0.875
Fever	45 (18.9)	17 (23.3)	0.514
Sore throat	44 (18.5)	15 (20.5)	0.824
Shot s of vaccine			0.009
1	137 (57.6)	55 (75.3)	
2	1 (0.4)	1 (1.4)	
3	47 (19.7)	7 (9.6)	
4	45 (18.9)	5 (6.8)	
5	8 (3.4)	5 (6.8)	
Blood test results			
Total protein (TP,g/l)	62.66 [60.03, 66.16]	60.73 [58.06, 63.60]	0.001
Albumin (g/l)	40.44 [38.77, 43.07]	39.07 [36.47, 40.46]	<0.001
Globulin (g/l)	21.11 [19.60, 23.10]	21.11 [19.90, 23.11]	0.646
A/G	1.90 [1.76, 2.07]	1.81 [1.64, 1.96]	0.003
Total bilirubin (umol/L)	11.50 [9.22, 15.68]	10.48 [7.94, 14.58]	0.154
Indirect bilirubin (umol/L)	16.00 [12.64, 24.68]	16.00 [12.43, 20.98]	0.660
Direct bilirubin (umol/L)	23.26 [19.36, 27.30]	24.26 [20.47, 29.38]	0.095
Urea (mmol/L)	5.36 [4.46, 6.26]	5.73 [4.76, 7.05]	0.004
Creatinine (mmol/L)	60.30 [51.73, 68.88]	66.70 [58.90, 88.00]	<0.001
Glomerular filtration rate (GFR,ml/min)	100.00 [92.00, 118.00]	92.00 [67.00, 105.00]	<0.001
kalium (mmol/L)	3.65 [3.51, 3.93]	3.65 [3.42, 3.79]	0.126
Sodium (Na) (mmol/L)	144.00 [143.00, 144.00]	144.00 [143.00, 144.00]	0.247
Chlorine (mmol/L)	106.00 [104.25, 107.00]	106.00 [103.00, 106.00]	0.054
Procalcitonin (ng/ml)	0.02 [0.02, 0.02]	0.02 [0.02, 0.04]	<0.001
Interleukin 6(IL-6) (pg/ml)	29.71 [13.14, 86.47]	29.71 [16.15, 127.40]	0.233
White blood cell (*10^9^/l,)	4.89 [4.12, 6.26]	4.76 [3.61, 5.41]	0.023
Red blood cell (*10^12^/l)	4.24 [3.97, 4.57]	3.98 [3.64, 4.45]	0.009
Hemoglobin (Hb) (g/l)	129.00 [121.00, 136.00]	121.00 [111.00, 133.00]	0.001
Hematocrit (%)	39.80 [37.30, 42.50]	37.70 [34.50, 41.20]	0.001
Mean corpuscular volume (MCV) (fl)	93.30 [90.43, 95.97]	93.00 [90.20, 96.50]	0.737
Mean corpuscular hemoglobin (MCH) (pg)	30.30 [29.22, 31.30]	30.30 [29.10, 31.40]	0.878
Mean corpuscular hemoglobin concentration (MCHC)(g/l)	324.00 [319.25, 329.00]	323.00 [319.00, 329.00]	0.490
Platelet (*10^9^/l)	170.00 [138.25, 225.75]	153.00 [125.00, 193.00]	0.013
Lymphocyte ratio (%)	28.60 [20.70, 36.18]	29.30 [22.20, 37.70]	0.420
Monocyte ratio (%)	8.50 [6.62, 10.47]	9.60 [7.60, 10.80]	0.057
Neutrophile granulocyte ratio (%)	60.87 (12.39)	58.67 (13.03)	0.191
Eosinophils ratio (%)	0.90 [0.40, 1.90]	0.80 [0.20, 1.70]	0.357
Red blood cell distribution width CV (%)	13.10 [12.70, 13.60]	13.10 [12.70, 13.50]	0.666
Red blood cell distribution width SD (fl)	44.70 [43.23, 46.70]	44.80 [42.80, 46.20]	0.574
Mean platelet volume (MPV) (fl)	10.20 [9.40, 11.00]	10.70 [9.50, 11.70]	0.004
Platelet distribution width (PDW) (fl)	16.20 [16.00, 16.50]	16.30 [16.10, 16.60]	0.029
Thrombocytocrit (%)	0.18 [0.15, 0.22]	0.16 [0.14, 0.20]	0.036
Platelet large cell ratio (P-LCR) (%)	27.40 [21.12, 32.48]	30.60 [23.50, 37.20]	0.004
Large platelet number (*10^9^/l)	46.00 [36.25, 58.75]	46.00 [36.00, 54.00]	0.555
C-reaction protein (mg/L)	4.76 [1.61, 9.70]	9.00 [5.61, 24.67]	<0.001
Serum amyloid protein (SAA,mg/L)	13.98 [7.36, 23.14]	13.98 [11.24, 58.70]	0.013

Compare of all the result of laboratory tests and chest CT and treatment of patients in the No Delirium and the Delirium groups.

The measurement variables are shown using mean and standard deviation (SD) OR the median and interquartile range, [IQR].

Count variables were shown as *n* (%).

^1^Days to the outcome: days to discharge (No Delirium group) or days to delirium (Delirium group).

We divided the chest computed tomogram into four degrees according to the reports: None, No *Pneumonia* or *Pulmonary interstitial hyperplasia* were shown in the report; *Pneumonia*, *Pneumonia* was shown in the report; *Pulmonary interstitial hyperplasia*, *Pulmonary interstitial hyperplasia* was shown in the report; *Pneumonia* and *Pulmonary interstitial hyperplasia*, both *Pneumonia* and *Pulmonary interstitial hyperplasia* were shown in the report. *p* < 0.05 was considered the difference is statistically significant.

**Figure 1:**
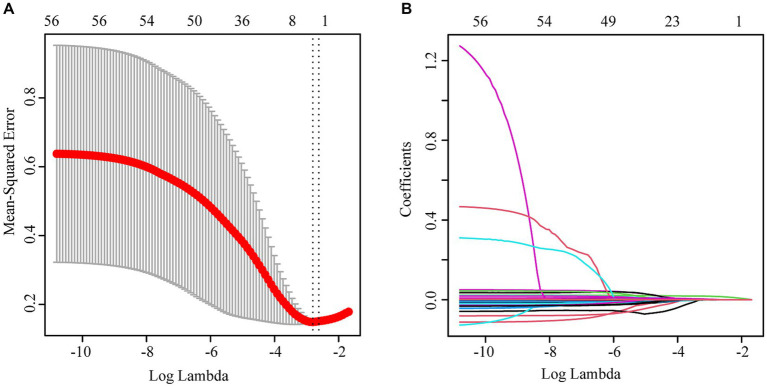
**(A)** lasso cross-validation curve; **(B)** Variable coefficient diagram of lasso regression model.

Three independent influencing factors of delirium were confirmed: age (OR1.16,1.11–1.22), Glomerular filtration rate (OR 0.98,0.97–0.99), platelet-large cell ratio (1.06,1.02–1.10), shown in Figure 1, Table 2.

**Table 2:** Results of univariate and multivariate logistic regression analysis with variants selected by lasso logistic regression.

Variables	Univariate regression		Multivariate regression	
	OR (95%CI)	*p* value	OR (95%CI)	*p* value
Age (y)	1.2 (1.1–1.2)	<0.001	1.16 (1.11, 1.22)	<0.001
GFR (ml/min)	0.98 (0.97–0.99)	<0.001	0.98 (0.97, 0.99]	0.012
P-LCR	1.1 (1–1.1)	0.002	1.06 (1.02, 1.10]	0.002

GFR, Glomerular filtration rate.

P-LCR, Platelet large cell ratio.

OR, odds ratios.

*p* < 0.05 was considered the difference is statistically significant.

**Figure 2:**
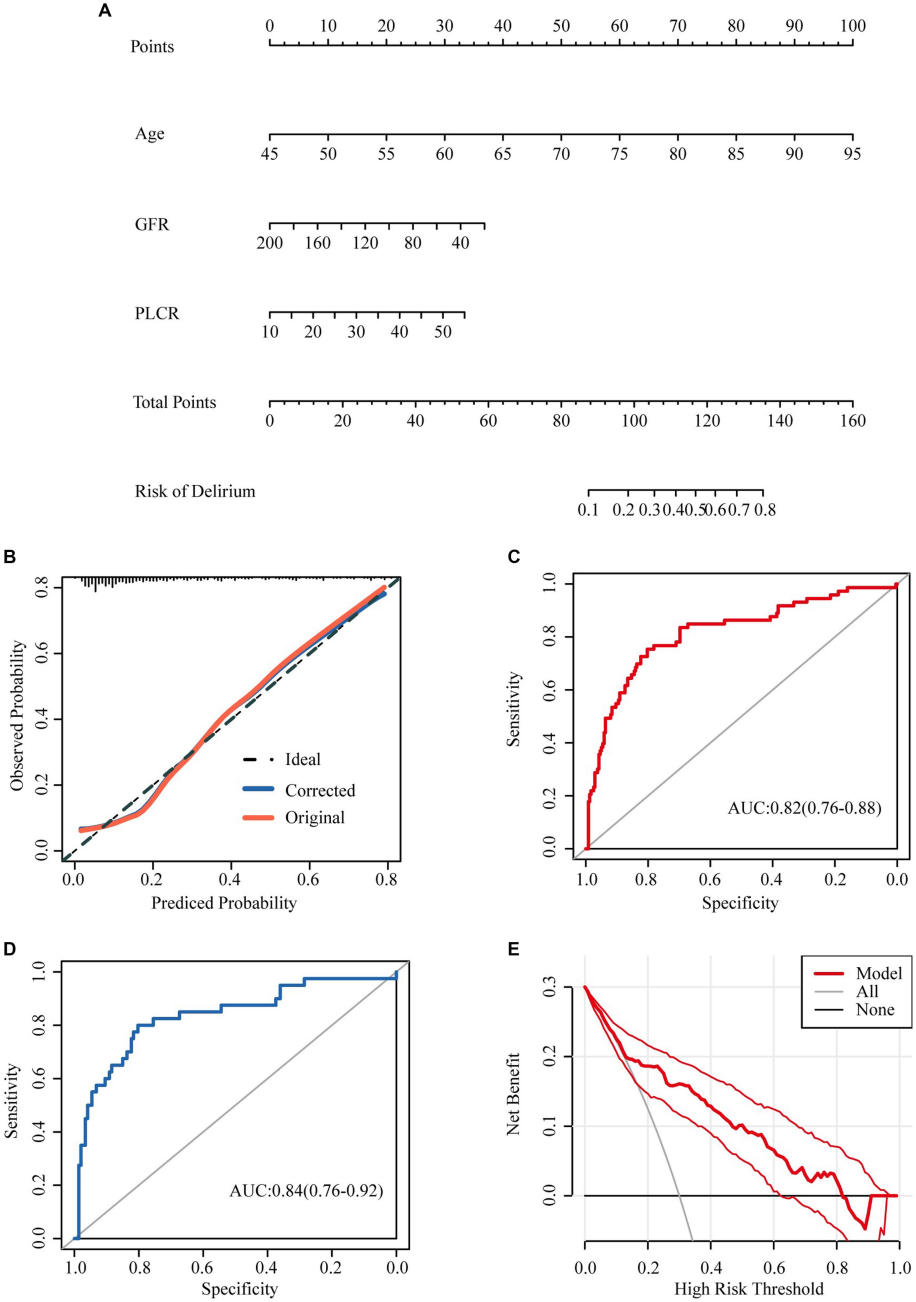
Nomograms predicting incidence of delirium in aged hospital patients with none-severe omicron infection. **(A)** Nomograms predicting incidence of delirium in aged hospital patients with none-severe omicron infection. **(B)** The calibration curve to analysis the predicted probability of the model. **(C)** The ROC curve was used to evaluate the predictive ability of the model. **(D)** 60% of the data were selected for validation to evaluate the predictive ability of the model. **(E)** The decision curve analysis was made to prove the validity of our prediction model.

Nomograms to predict delirium in elderly patients are shown in Figure 2A. The nomogram was created based on the following independent factors: age, GFR, and P-LCR. Higher total points were associated with an increased probability of delirium development.

The calibration curve showed that the calibration curve fitted the ideal curve calibration well (Figure 2B), indicating that the predicted probability of the model was consistent with the actual probability, and the model had a good calibration.

The ROC curve was used to evaluate the predictive ability of the model, and the AUC was 0.82 (0.76–0.88) (Figure 2C). 60% of the data were selected for validation to evaluate the predictive ability of the model, with an AUC of 0.84 (0.76–0.92) (Figure 2D).

The decision curve analysis was made to prove the validity of our prediction model (Figure 2E). The analysis showed that prediction model was useful while the incidence ranges from 0.15–0.82.

## Discussion

In this study, we studied the incidence of delirium among hospitalized elderly patients with non-severe SARS-CoV-2 infection caused by the Omicron variant, during the periods when it was the dominant variant in Shanghai. Previous studies have shown that cognitive impairment is common among patients with COVID-19, including those with mild disease (15), and that this may persist for a long term, taking a toll on the patient, the society, and the economy (17, 18). Therefore, despite the reduced pathogenicity of Omicron (6, 7), resulting in mostly mild disease, the cognitive impairment among these patients is still noteworthy due to the large numbers of overall infections and elderly patients infected; baseline cognitive impairment among elderly patients is also very common. Our study showed that 23.47% of elderly patients with non-severe Omicron infection developed delirium while admitted. Studies also showed that impaired hippocampal neurogenesis, decreased oligodendrocytes and myelin loss in subcortical white matter were evident at 1 week, and persisted until at least 7 weeks, following mild respiratory SARS-CoV-2 infection (14) and may lead to long-term cognitive decline (15).

We confirmed three independent influencing factors of delirium in our study: age (OR1.16,1.11–1.22), Glomerular filtration rate (OR 0.98,0.97–0.99), platelet-large cell ratio (1.06,1.02–1.10).Age is a well-known risk factor (19), even in non-severe illness among older adults infected with Omicron (OR 1.16, 95% CI 1.11–1.22, *p* < 0.001). Delirium increased by 0.16 times with each one-year increase in age.

Kidney involvement is frequent in covid-19 (20, 21), acute kidney injury is a risk factor for delirium and coma during critical illness (22), studies showed that Delirium was associated with a lower eGFR (23, 24).Our study also proved that the GFR was an independent influencing factors of delirium, which is consistent with previous studies. Renal damage caused by SARS-CoV-2 was multifactorial, it can directly infect renal podocytes and proximal tubular cells and cause acute tubular necrosis, Bowman’s capsule protein leakage, glomerular collapse disease, and mitochondrial damage based on the angiotensin-converting enzyme 2 (ACE2) pathway (25).

Platelet levels indicate inflammation, especially pulmonary inflammation, such as in COVID-19 (26), and prothrombotic responses in many viral infections. Multiple factors activate platelets in COVID-19, resulting in their depletion (27), as indicated by an increased P-LCR. Large platelets are more metabolically and enzymatically active and produce more procoagulants, vasoactive and adhesive factors; thus, they promote thrombosis and atherosclerosis (28). Studies showed that P-LCR increases in COVID-19 patients (29) and increased P-LCR is associated with poor prognosis (30), which parallels our results showing a positive association with severe inflammation, which itself is associated with delirium (31).

Finally, we construct a nomogram to predict the incidence of delirium in elderly patients with non-severe SARS-CoV-2 infection based on age, GFR, and P-LCR. Higher total points were associated with an increased probability of delirium development. For example, a patient aged 75 years (60 points) with a GFR 60 (30 points), P-LCR of 50 (30 points) would have a total of 120 points, for a predicted incidence 50–60%. The ROC curve was used to evaluate the predictive ability of the model, and the AUC was 0.82 (0.76–0.88), indicating that the prediction of the model was very accurate (Figure 2C). And then 60% of the data were selected for validation to evaluate the predictive ability of the model, with an AUC of 0.84 (0.76–0.92) (Figure 2D).The predictive ability of the model evaluated by internal validation suggested that the predictive ability of the model was good.

The decision curve analysis was made to prove the validity of our prediction model (Figure 2E). The analysis showed that prediction model was useful while the incidence ranges from 0.15–0.82. Hence, we can conclude that our model can be used for clinicians to predict the onset of delirium in patients, so as to achieve timely prevention, diagnosis and treatment. There are no definitive medications to prevent the development of delirium, Some humanistic measures may be effective: delivering holistic care with a ‘home-like’ (H) environment, more time with the family (32), Active treatment of the COVID-19 to prevent nonsevere cases progressing to severe cases, study had indeed showed that delirium is especially common in those with severe disease on hospital admission (33).

In summary, 311 patients’ information were used to construct a prediction model by nomogram for delirium in elderly patients during hospitalization with Non-severe SARS-CoV-2 infection. Through internal verification, we proved that the model has a good predictive effect. Selecting variables by lasso regression has the advantages of simplifying the statistical model, reducing multivariate collinearity and improving the accuracy of the model. This is the first large-scale clinical data to predict the risk of delirium during hospitalization in patients with mild infection. While our limitation was that no external validation data was used for validation.

## Data availability statement

The original contributions presented in the study are included in the article/supplementary material, further inquiries can be directed to the corresponding authors.

## Ethics statement

The studies involving humans were approved by the protocol was reviewed and approved by the Medical Ethics Committee of Shanghai Fourth Hospital. The studies were conducted in accordance with the local legislation and institutional requirements. The participants provided their written informed consent to participate in this study.

## Author contributions

GA: Conceptualization, Data curation, Formal analysis, Funding acquisition, Investigation, Methodology, Validation, Writing – original draft. ZM: Conceptualization, Data curation, Investigation, Methodology, Resources, Writing – original draft. DH: Investigation, Writing – review & editing. DO: Investigation, Writing – review & editing. XC: Investigation, Writing – review & editing. QL: Supervision, Validation, Writing – review & editing. LX: Project administration, Supervision, Visualization, Writing – review & editing. CL: Methodology, Project administration, Supervision, Validation, Writing – review & editing.
